# Characterization of signaling pathways regulating the expression of pro-inflammatory long form thymic stromal lymphopoietin upon human metapneumovirus infection

**DOI:** 10.1038/s41598-018-19225-0

**Published:** 2018-01-17

**Authors:** Youxian Li, Cecilie Lund, Ida Nervik, Simon Loevenich, Henrik Døllner, Marit W. Anthonsen, Ingvild B. Johnsen

**Affiliations:** 1 Department of Clinical and Molecular Medicine, Faculty of Medicine and Health Science, Norwegian University of Science and Technology, Trondheim, 7491 Norway; 2 Children’s Department, St. Olavs University Hospital, Trondheim, 7030 Norway

## Abstract

Thymic stromal lymphopoietin (TSLP) is associated with several allergic diseases including asthma. Two isoforms of TSLP exist in humans, a long form (lfTSLP) and a short form (sfTSLP), displaying distinct immunological functions. Recently, TSLP was found to be upregulated in human airway cells upon human metapneumovirus (hMPV) infection, yet it remains unclear if the two isoforms are regulated differently during hMPV infection. Importantly, the molecular mechanisms underlying hMPV-mediated TSLP induction remain undescribed. In this study, we characterized the expression and regulation of TSLP in hMPV-infected human airway cells. We demonstrated that hMPV strongly induced the expression of pro-inflammatory lfTSLP in human airway epithelial cells and lung fibroblasts. Further, knockdown of pattern recognition receptors retinoic acid-inducible gene I (RIG-I) or Toll-like receptor 3 (TLR3), as well as downstream signal transducers, abrogated hMPV-mediated lfTSLP induction. Importantly, silencing of TANK-binding kinase 1 (TBK1) also impaired hMPV-mediated lfTSLP induction, which could be attributed to compromised NF-κB activation. Overall, these results suggest that TBK1 may be instrumental for hMPV-mediated activation of NF-κB downstream RIG-I and TLR3, leading to a specific induction of lfTSLP in hMPV-infected human airway cells.

## Introduction

Respiratory tract infections (RTIs) are a major cause of childhood mortality and morbidity worldwide, especially in developing countries^1^. Human metapneumovirus (hMPV), first isolated in 2001^2^, is considered one of the leading causes of hospitalization for RTIs among children <5 years of age^3,4^. hMPV is an enveloped virus with a single-stranded, negative-sense RNA genome and belongs to the Paramyxoviridae family. hMPV primarily replicates in the epithelial cells in the upper and lower respiratory tract. Upon infection the viral RNA genome is believed to be delivered into the cytosol where the replication cycle initiates^5^. Host cells are equipped with pattern recognition receptors (PRRs), including membrane bound Toll-like receptors (TLRs) and cytoplasmic RIG-I-like receptors (RLRs), which recognize pathogen associated molecular patterns (PAMPs), such as viral RNA. Recognition of PAMPs triggers signaling cascades that typically involve IκB kinase (IKK) or IKK-related kinases, such as TANK-binding kinase 1 (TBK1), leading to activation of transcription factors and transcriptional upregulation of cytokines and chemokines that in turn regulate the inflammatory responses in the infected host^6^.

Interestingly, RTIs in the first years of life are associated with later asthma development^7^. Many viruses and bacteria can trigger similar asthmatic symptoms in young children^8^. Recently it has been proposed that susceptibility and host immune response to respiratory tract infections in general, rather than the particular triggers, are the important risk factors for asthma^9^. Thymic stromal lymphopoietin (TSLP), an interleukin 7 (IL-7)-like cytokine associated with several allergic diseases including asthma, has been suggested to be a liaison between RTIs and asthma development^10,11^. TSLP was found to be overexpressed in the airways of asthmatic patients^12,13^. Studies in mouse models of asthma have clearly demonstrated that TSLP is a key initiator of allergic airway inflammation^14,15^. Importantly, TSLP has been shown to be upregulated in human airway epithelial cells upon infection with respiratory viruses, such as rhinovirus (RV), respiratory syncytial virus (RSV) and hMPV, providing a possible link between viral infections and asthma pathogenesis^16–18^. TSLP acts on multiple cell types including dendritic cells (DCs), lymphocytes and granulocytes^19^. In particular, TSLP-conditioned DCs prime naïve T cells to differentiate into a T-helper type 2 (Th2) phenotype, producing cytokines involved in allergic inflammation including IL-4, IL-5, IL-13, and TNF-α^20,21^. Two isoforms of TSLP, a long form (lfTSLP) and an alternative short form (sfTSLP), are present in humans. Recent studies suggest that lfTSLP is induced by pro-inflammatory signals and associated with allergic inflammation, whilst sfTSLP is constitutively expressed and may have an anti-inflammatory or anti-microbial role^22–24^. In this study, we examined the differential expression of lfTSLP and sfTSLP in human airway epithelial cells and lung fibroblasts upon hMPV infection. We further investigated the signaling pathways mediating hMPV-induced lfTSLP expression using human lung fibroblasts as the model system.

## Results

### hMPV infection strongly induces lfTSLP expression in human airway epithelial cells and lung fibroblasts

**Figure 1:**
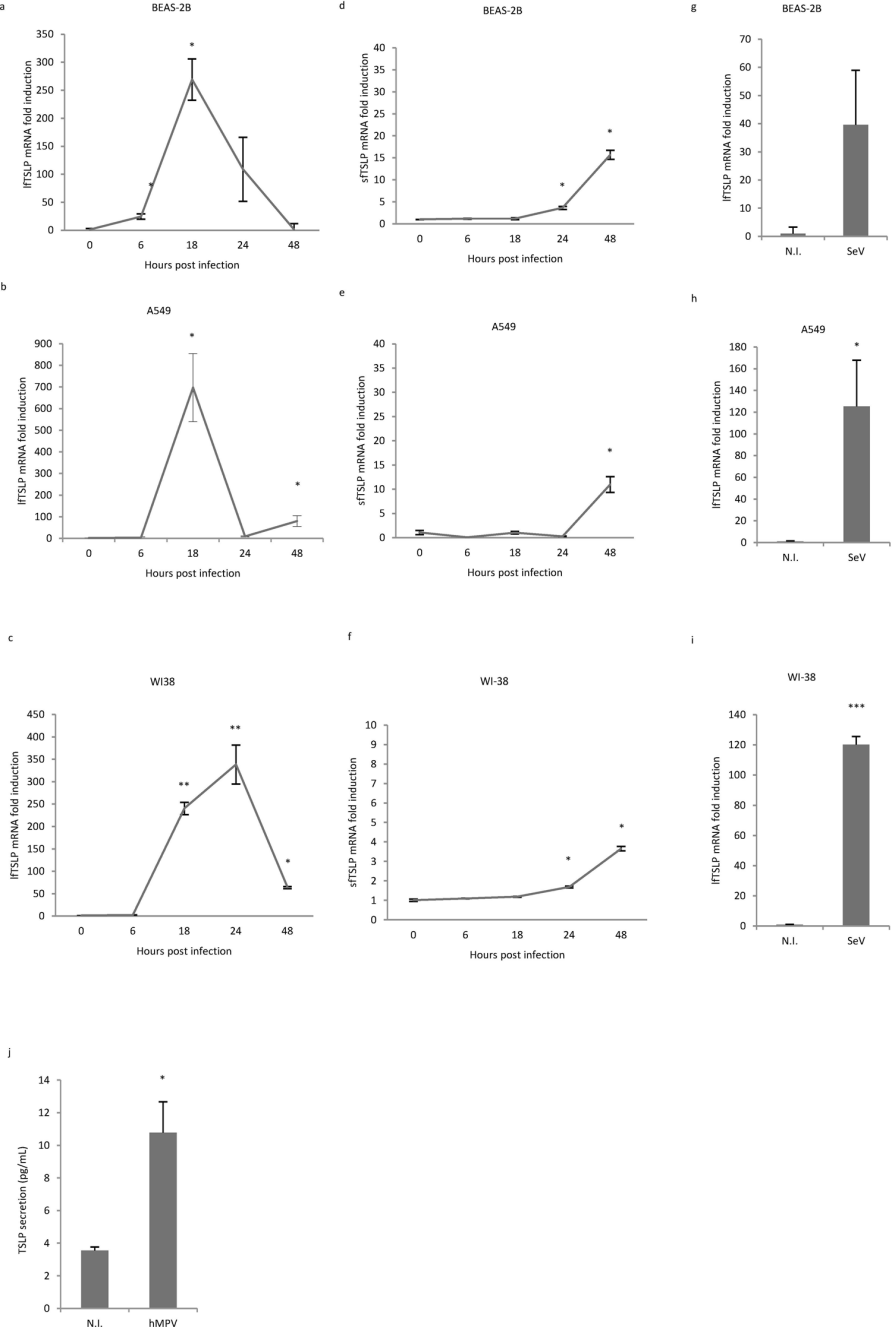
hMPV infection triggers a robust induction of lfTSLP in human airway epithelial cells and lung fibroblasts. (**a**–**i**) BEAS-2B, A549 and WI-38 cells were infected with hMPV(**a**–**f**) at indicated time points, or with SeV(g-i) for 24 hours. lfTSLP (**a**–**c**, **g**–**i**) and sfTSLP (**d**–**f**) mRNA expression was assessed by qRT-PCR. (**j**) WI-38 cells were infected with hMPV for 24 hours. Supernatants were collected and TSLP protein secretion was measured by ELISA. Data are representative for at least 3 independent experiments. Error bars represent SD of 3 technical replicates. *p < 0.05, **p < 0.01, ***p < 0.001. (Compared to non-infected samples - N.I.).

It has been previously demonstrated that hMPV induces TSLP transcription in human alveolar epithelial cells^18^. To confirm this finding, and importantly, to examine if the two TSLP isoforms are differentially expressed during hMPV infection, we assessed the gene expression of lfTSLP and sfTSLP in three different cell lines derived from human respiratory system: two human airway epithelial cell lines (BEAS-2B and A549) and a human fetal lung fibroblast cell line (WI-38). The cell lines were chosen because of their high susceptibility towards hMPV infection, as evidenced by rapid hMPV replication in all three cell lines (Supplementary Fig. 1). Cells were infected by hMPV at multiplicity of infection (MOI) 1 and mRNA expression levels of lfTSLP and sfTSLP were determined by quantitative real-time PCR (qRT-PCR). GAPDH, which has been shown to be reliable under viral-infection conditions^25^, was used as the reference gene for normalization. hMPV infection induced a robust upregulation of lfTSLP mRNA expression in both airway epithelial cells (Fig. 1a,b) and fibroblasts (Fig. 1c), peaking at 18 h or 24 h post infection. In contrast, only a modest and delayed increase of sfTSLP mRNA expression was observed (Fig. 1d–f). Sendai virus, a related *Paramyxoviridae* member which has been shown to induce TSLP expression in human airway epithelial cells *in vitro*^17^, was used as a positive control. Infection of Sendai virus also strongly induced lfTSLP expression at 24 h (Fig. 1g–i). In addition, hMPV infection resulted in increased TSLP secretion in WI-38 cells (Fig. 1j). Commercially available antibodies prepared with recombinant full-length TSLP recognize both isoforms, as the sfTSLP protein sequence completely overlaps with the lfTSLP sequence in the C-terminal region^24^. Hence, we were unable to differentiate the 2 isoforms at the protein level. Taken together, our qRT-PCR results demonstrate that hMPV infection leads to a prominent induction of pro-inflammatory lfTSLP in human airway cells.

### hMPV-mediated induction of lfTSLP is dependent on viral replication

**Figure 2:**
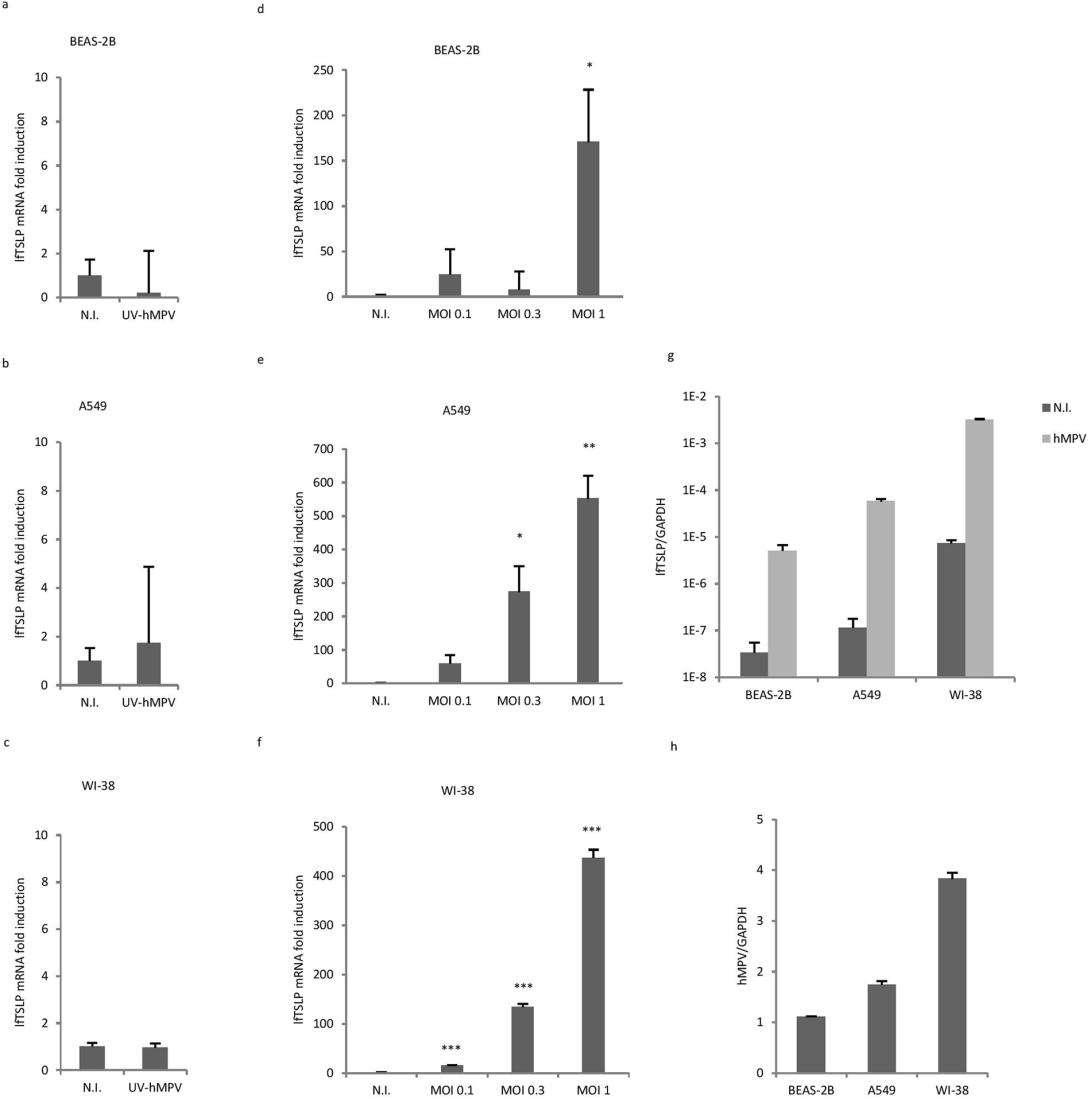
The induction of lfTSLP in human airway cells is dependent on hMPV replication. (**a**–**c**) BEAS-2B (**a**), A549 (**b**) and WI-38 (**c**) cells were infected with UV-inactivated hMPV or wild type hMPV for 18 hours. lfTSLP mRNA expression was assessed by qRT-PCR. (d-f) BEAS-2B (**d**), A549 (**e**) and WI-38 (**f**) cells were infected with hMPV at indicated MOIs for 18 hours. lfTSLP mRNA expression was assessed by qRT-PCR. (**g**,**h**) Cells were infected with hMPV for 18 hours, lfTSLP mRNA expression (**g**) and hMPV viral RNA expression (**h**) was compared to GAPDH expression by qRT-PCR. Data are representative for at least 3 independent experiments. Error bars represent SD of 3 technical replicates. *p < 0.05, **p < 0.01, ***p < 0.001. (Compared to non-infected samples - N.I.).

Following membrane fusion of hMPV with host cells, the negative-sense viral RNA (vRNA) genome is released into the cytoplasm, serving as the template for the synthesis of mRNA and antigenomic cRNA^26^. To evaluate if hMPV-mediated induction of lfTSLP is dependent on viral replication we exposed A549, BEAS-2B and WI-38 cells to UV-inactivated hMPV. UV-inactivation effectively abrogated hMPV replication (Supplementary Fig. 2a) and hMPV-induced interferon-β expression (Supplementary Fig. 2b). UV-inactivated hMPV also failed to induce lfTSLP (Fig. 2a–c), indicating that host cells recognize hMPV replication intermediates, presumably dsRNA, to mount a lfTSLP response. As expected, cells infected with different MOIs of hMPV showed a dose-dependent induction of lfTSLP (Fig. 2d–f). Finally, we observed that the expression of lfTSLP was relatively stronger in WI-38 cells than in A549 or BEAS-2B cells, as demonstrated by directly comparing the mRNA expression of lfTSLP to that of GAPDH (Fig. 2g). The difference in lfTSLP expression between the cell lines could be explained by different hMPV replication rates, as shown in Fig. 2h that more viral RNA accumulated in WI-38 cells than in BEAS-2B or A549 cells. Immunofluorescence imaging of infected cells stained with hMPV N-protein also revealed higher viral yield in WI-38 cells than in A549 cells, despite that both cells were nearly 100% infected (Supplementary Fig. 3). Taken together these results indicate that the induction of lfTSLP is dependent on hMPV replication.

### RIG-I and TLR3 signaling pathways mediate lfTSLP expression in hMPV-infected airway cells

**Figure 3:**
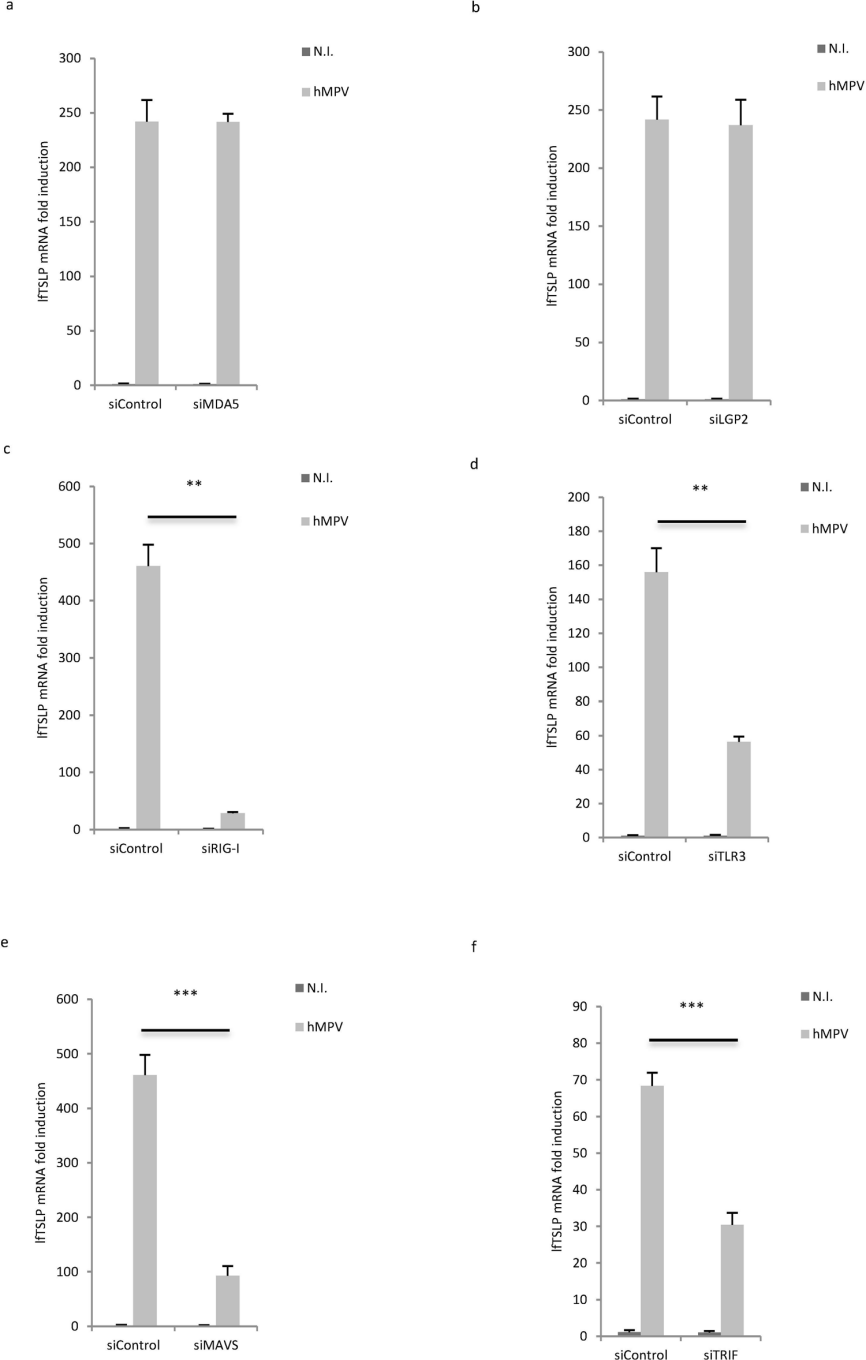
RIG-I – MAVS and TLR3 – TRIF signaling is required for hMPV-mediated lfTSLP induction. WI-38 cells were transfected with siRNAs against MDA5 (**a**), LGP2 (**b**), RIG-I (**c**), TLR3 (**d**), MAVS (**e**), TRIF (**f**) or control siRNA (siControl) prior to infection with hMPV for 18 hours. lfTSLP mRNA expression was assessed by qRT-PCR. Data are representative for at least 3 independent experiments. Error bars represent SD of 3 technical replicates. **p < 0.01, ***p < 0.001.

The molecular mechanisms behind hMPV-mediated induction of TSLP have not been described. As our results demonstrated a robust induction of pro-inflammatory lfTSLP upon hMPV infection, we next sought to determine the PRRs that sense hMPV replication leading to lfTSLP expression using WI-38 cells as the model system. WI-38 cells were transfected with siRNAs targeting the cytoplasmic RNA sensors RIG-I, melanoma differentiation-associated protein 5 (MDA5) and the laboratory of genetics and physiology 2 (LGP2) (Fig. 3a–c), as well as the endosomal dsRNA sensor TLR3 (Fig. 3d) prior to hMPV infection. siRNA-mediated knockdown of both RIG-I and TLR3 inhibited hMPV-mediated lfTSLP induction (Fig. 3c,d), whilst knockdown of MDA5 or LGP2 had no inhibitory effect (Fig. 3a,b). In accordance with these data, knockdown of mitochondrial antiviral signaling protein (MAVS) and TIR-domain-containing adapter-inducing interferon-β (TRIF), the adaptor proteins of RIG-I and TLR3 respectively, also suppressed hMPV-mediated lfTSLP induction (Fig. 3e,f). Silencing efficiency of siRNAs was validated by qRT-PCR (Supplementary Fig. 4a–f). Since activation of PRRs such as RIG-I and TLR3 could also lead to production of type I or type III interferons^6,27^, we further assessed if type I or type III interferons induce lfTSLP. As shown in Supplementary Fig. 5a, in contrast to hMPV infection, treatment of recombinant interferon-β (rIFNβ) alone did not induce lfTSLP, suggesting that lfTSLP is not an interferon-inducible gene. As the positive control both rIFNβ treatment and hMPV infection lead to increased expression of 2′–5′-Oligoadenylate Synthetase 3 (OAS3), an established interferon-inducible gene^28^ (Supplementary Fig. 5b). On the other hand, WI-38 cells did not respond to recombinant interferon-λ1 (rIFNλ1); neither lfTSLP nor the two interferon inducible genes tested (OAS3 and ISG54) were induced by rIFNλ1 treatment (Supplementary Fig. 5c–e), suggesting that WI-38 cells may not express type III interferon receptors and that type III interferons were irrelevant to lfTSLP induction at least in this setting. The same rIFNλ1 treatment strongly induced OAS3 and ISG54 expression in human monocyte-derived macrophages (Supplementary Fig. 5f,g), confirming the bioactivity of rIFNλ1. Overall, these data demonstrate that both RIG-I and TLR3 regulate the expression of pro-inflammatory lfTSLP upon hMPV infection in human airway cells.

### TBK1 is required for hMPV-mediated induction of lfTSLP

**Figure 4:**
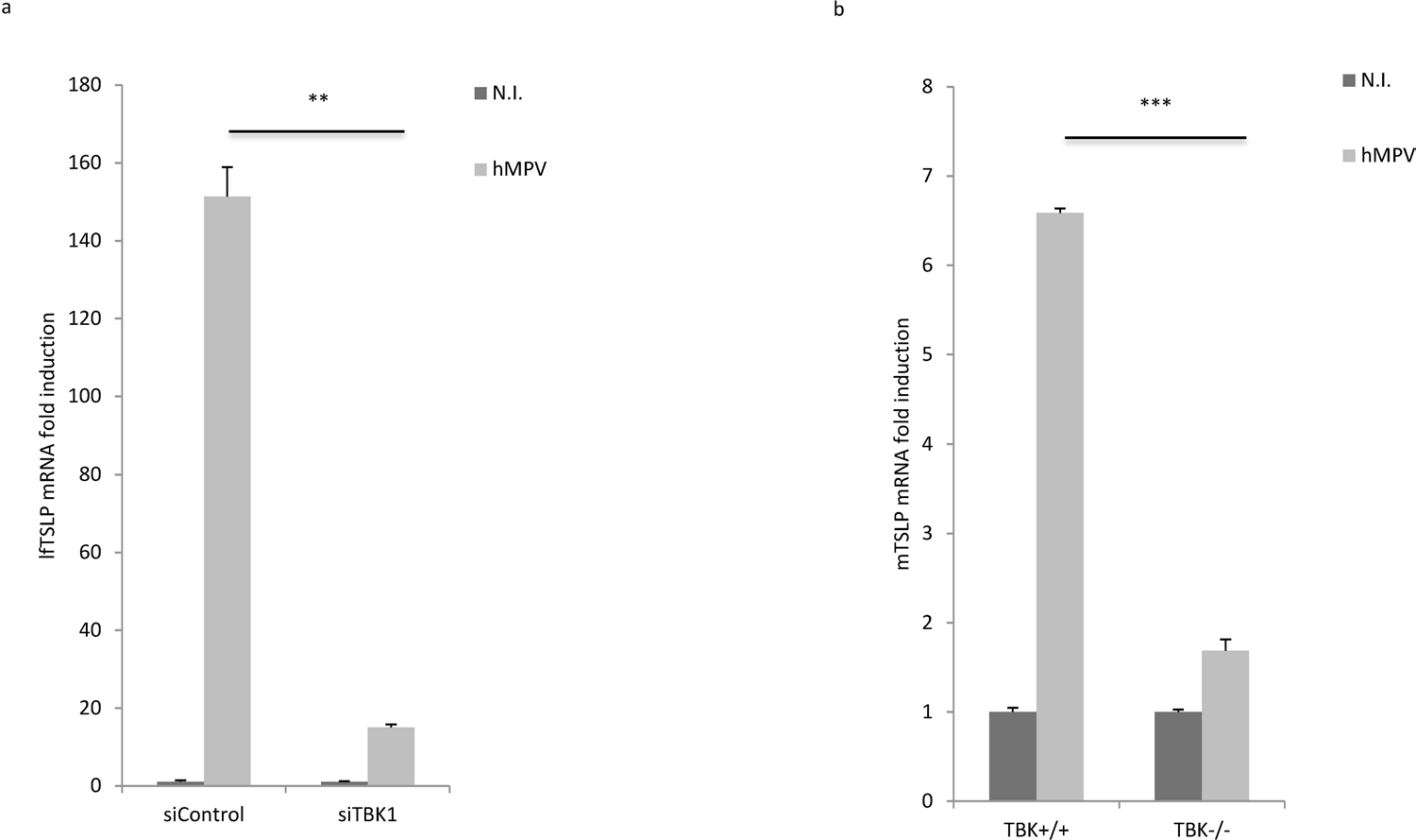
TBK1 is required for hMPV-mediated induction of lfTSLP. (**a**) WI-38 cells were transfected with siRNAs against TBK1 or control siRNA (siControl) prior to infection with hMPV for 18 hours. lfTSLP mRNA expression was assessed by qRT-PCR. (**b**) Immortalized TBK1 +/+ or TBK1 −/− MEF cells were infected with hMPV for 24 hours. Mouse TSLP (mTSLP) mRNA expression was assessed by qRT-PCR. Data are representative for at least 3 independent experiments. Error bars represent SD of 3 technical replicates. **p < 0.01, ***p < 0.001.

While the RIG-I and TLR3 pathways utilize distinct adaptor proteins, many of the signaling events downstream these PRRs converge at the level of the IKK and IKK-related kinases^29^. Several studies have demonstrated that the IKK-related kinases, TBK1 in particular, are crucial components of the antiviral PRR pathways^30–33^. Hence we went on to examine if TBK1 is involved in hMPV induced lfTSLP expression. Knockdown studies showed that silencing of TBK1 inhibited hMPV-mediated lfTSLP induction (Fig. 4a). Silencing efficiency of TBK1 siRNA was validated by qRT-PCR (Supplementary Fig. 4g). To further confirm the role of TBK1 in hMPV-mediated lfTSLP induction, we infected immortalized murine embryonic fibroblast (MEF) cells derived from wild type (TBK1 +/+) or TBK1 knockout (TBK1 −/−) mice with hMPV. In accordance with the knockdown experiment, hMPV-induced mouse TSLP mRNA expression was impaired in TBK1^−/−^ MEF cells compared to that in TBK1 +/+MEF cells (Fig. 4b). Taken together, these data demonstrate that TBK1 is crucial for the induction of lfTSLP during hMPV infection.

### hMPV-mediated lfTSLP expression is dependent on NF-κB activation (through TBK1)

**Figure 5:**
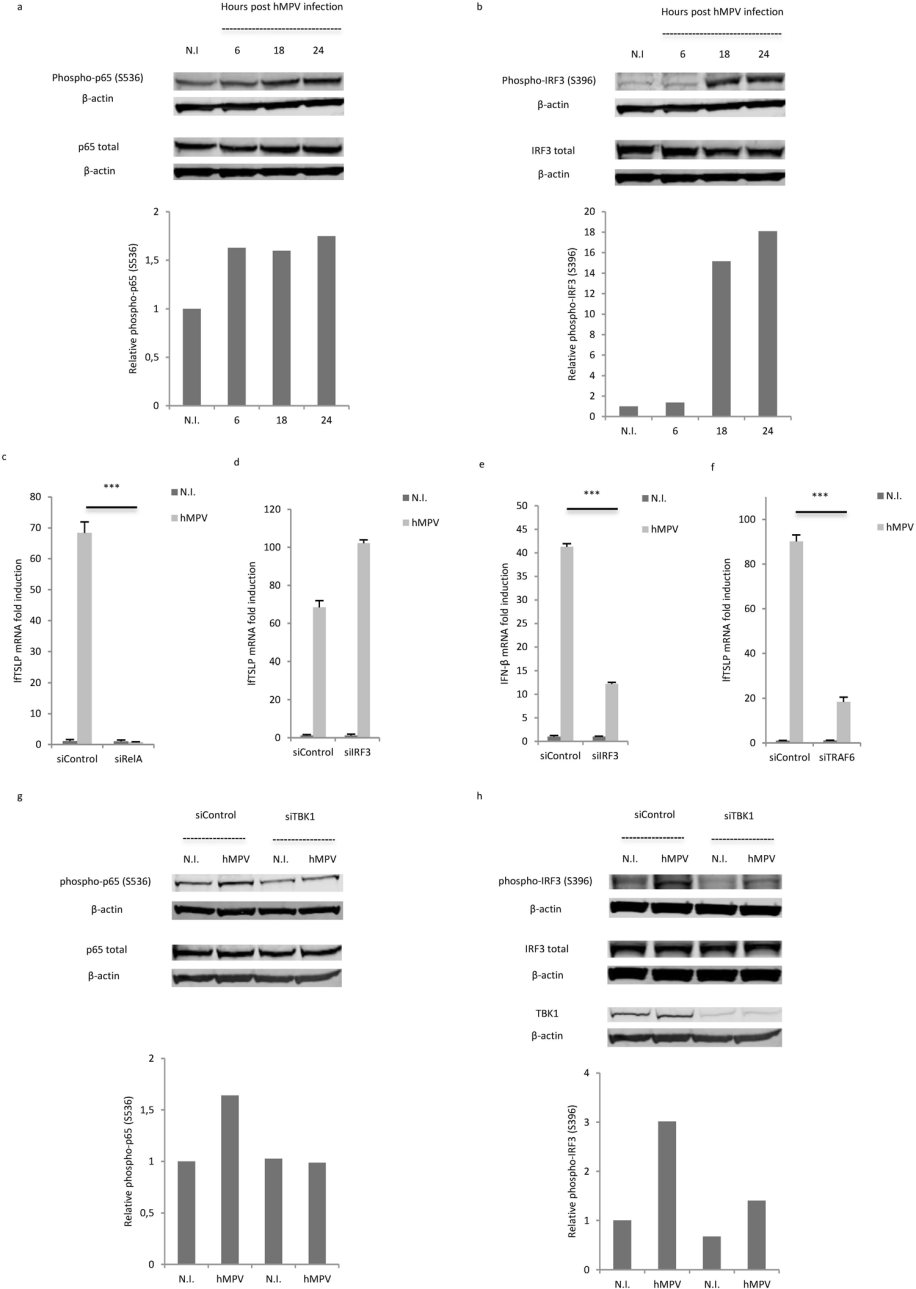
hMPV-mediated lfTSLP induction is dependent on TBK1-mediated NF-κB activation. (a, b) WI-38 cells were infected with hMPV and cells were lysed at indicated time points post hMPV infection. Phosphorylation of the NF-κB p65 subunit (**a**) and IRF3 (**b**) was analyzed by Western Blot. Lower panels: Relative phosphorylation levels were calculated by normalizing phospho-p65 (S536) or phospho-IRF3 (S396) against total-p65 or total-IRF3 respectively (each normalized against β-actin first). Data are representative for at least 3 independent experiments. (**c**–**f**) WI-38 cells were transfected with siRNAs against RelA (**c**), IRF3 (**d**,**e**), TRAF6 (**f**) or control siRNA (siControl) prior to infection with hMPV for 18 hours. lfTSLP (**c**,**d**,**f**) and IFN-β (**e**) mRNA expression was assessed by qRT-PCR. (**g**,**h**) WI-38 cells were transfected with siRNAs against TBK1 or control siRNA prior to infection with hMPV for 24 hours. Cells were lysed and phosphorylation of the NF-κB p65 subunit (**g**) and IRF3 (**h**) was analyzed by Western blot. Lower panels: Relative phosphorylation levels were calculated in the same manner as (**a**,**b**). Full-length blots are presented in Supplementary Information. Data are representative for at least 3 independent experiments. Error bars represent SD of 3 technical replicates. ***p < 0.001.

Binding of viral RNA to RIG-I or TLR3 activates signaling cascades leading to phosphorylation of transcription factors NF-κB and IRF-3, and subsequent transcriptional regulation of pro-inflammatory cytokines and type I interferons^6^. NF-κB was shown to be important for inflammatory cytokine-, dsRNA- or Sendai virus-mediated TSLP induction^34,35,16,17^. IRF-3 was also suggested to be involved in TSLP induction mediated by dsRNA or microbiota-derived nucleic acids^16,36^. To elucidate the transcription factors behind hMPV-mediated lfTSLP induction, we first sought to determine if hMPV infection induces phosphorylation of NF-κB and IRF3 in human airway cells. In line with what was reported in human airway epithelial cells^37^, Western blot analysis revealed increased levels of phosphorylation of both NF-κB and IRF-3 in WI-38 cells 18 h and 24 h after hMPV infection, as assessed by monitoring NF-κB p65 S536 phosphorylation and IRF3 S396 phosphorylation respectively (Fig. 5a,b). This indicates that both NF-κB and IRF3 are activated by hMPV infection. To further assess if these transcription factors specifically regulate lfTSLP transcription in hMPV-infected cells, we treated WI-38 cells with siRNAs targeting RelA (which encodes the NF-κB p65 subunit) or IRF3 prior to infection. siRNA-mediated knockdown of RelA almost completely abrogated hMPV-triggered induction of lfTSLP (Fig. 5c), suggesting a prominent role of NF-κB in the regulation of lfTSLP transcription in hMPV-infected cells. In contrast, silencing of IRF3 showed no inhibitory effect (Fig. 5d). This suggests that IRF3 is not required for hMPV-mediated lfTSLP induction. As a positive control, silencing of IRF3 resulted in reduced interferon-β expression upon hMPV infection (Fig. 5e). Moreover, siRNA-mediated knockdown of TNF receptor associated factor 6 (TRAF6), the ubiquitin E3 ligase that plays a pivotal role in NF-κB activation^38^, also inhibited hMPV-mediated lfTSLP induction (Fig. 5f), further verifying the importance of NF-κB activation in hMPV-mediated lfTSLP induction. Silencing efficiency was validated by qRT-PCR (Supplementary Fig. 4h–j).

Since our results show that TBK1 is crucial for hMPV-mediated lfTSLP induction, whereas IRF3 is not, we finally sought to assess if TBK1 regulates the activation of NF-κB during hMPV infection. Interestingly, we found that knockdown of TBK1 abrogated hMPV-triggered increase of phosphorylation of p65 in WI-38 cells (Fig. 5g), suggesting that TBK1 may act upstream of NF-κB signaling to mediate the induction of lfTSLP in this context. As expected knockdown of TBK1 impaired TBK1 protein expression itself, as well as hMPV-induced IRF3 phosphorylation (Fig. 5h). Taken together, our results show that NF-κB is crucial for the transcriptional regulation of lfTSLP through TBK1 in hMPV infected human airway cells.

## Discussion

The dichotomy of lfTSLP and sfTSLP had not been emphasized until recently, when it was proposed that the two isoforms may have distinct or even opposite functions^23,24^. lfTSLP is capable of activating DCs through binding to the receptor complex formed by thymic stromal lymphopoietin receptor (TSLPR) and the IL-7 receptor α chain, and inducing phosphorylation of signal transducer and activator of transcription 5 (STAT5), whereas sfTSLP does not induce STAT5 phosphorylation in DCs^23,24^. Instead sfTSLP seems to have an anti-inflammatory effect by inhibiting cytokine production in DCs^23^, and has been shown to protect airway epithelial barrier from allergen-induced disruption^39^. sfTSLP has also been suggested to be an antimicrobial peptide produced in the oral cavity and on the skin^24^. In this study, we show that hMPV infection strongly induces the pro-inflammatory isoform lfTSLP, both in human airway epithelial cells and lung fibroblasts, whereas sfTSLP expression is only slightly upregulated upon hMPV infection. Our results are consistent with what has been reported earlier that lfTSLP is strongly induced by inflammatory stimuli, whilst sfTSLP seems to be constitutively expressed^22–24,40^. The specific strong induction of pro-inflammatory lfTSLP in hMPV-infected airway cells may contribute to a microenvironment that favors Th2 responses. We also notice that the slight increase of sfTSLP only occurs at later time points after hMPV infection. Whether this is a negative feedback loop to bring the inflammatory response back to control, owing to the anti-inflammatory property of sfTSLP, needs further investigation.

TSLP is proposed to be primarily expressed by epithelial cells at barrier surfaces^19^. However other cell types have also been reported to produce TSLP, including dendritic cells, smooth muscle cells, and lung fibroblasts^41–43^. We found that the expression of lfTSLP is higher in the fibroblast cell line WI-38 cells compared to that in airway epithelial cell lines during hMPV infection, possibly due to the higher viral replication rate in WI-38 cells. This makes WI-38 cells a good model system to investigate the molecular mechanisms modulating hMPV-mediated lfTSLP induction. It also suggests that lung fibroblasts might be a relevant source of lfTSLP under pathophysiological conditions, especially when the epithelial barrier is damaged and interstitial fibroblasts are exposed to virus or other stimulants.

Our knockdown studies clearly demonstrate that both RIG-I- and TLR3-dependent signaling pathways contribute to hMPV-mediated lfTSLP induction. In addition, NF-κB activation is shown to be critical to lfTSLP induction downstream RIG-I and TLR3 activation, as knockdown of RelA almost completely abolishes hMPV-mediated lfTSLP expression, whilst knockdown of IRF3 shows no repressive effect. These results are consistent with a previous report showing that RSV-mediated TSLP induction is dependent on RIG-I, and that activation of NF-κB, but not IRF3 or IRF7, mediates SeV-induced TSLP^17^.

TBK1 was initially identified in the context of its ability to regulate NF-κB *in vitro*^44,45^, yet later studies in TBK1 deficient mice demonstrated that TBK1 is an essential component of the IRF3 signaling pathway after both viral infection and stimulation of TLR3 by dsRNA^31^. Later it was reported that TBK1 is not required for NF-κB activation during TLR3/4 stimulation or Sendai virus infection^33^. More recently, it was demonstrated that herpes simplex virus-1 (HSV-1)-dependent pro-inflammatory gene induction necessitates NF-kB activation through TBK1^46^. Altogether, it is likely that the involvement of TBK1 in NF-κB activation and downstream cytokine induction is context dependent. We show that both hMPV-triggered NF-kB phosphorylation and hMPV-mediated lfTSLP induction are hampered when TBK1 is knocked down in human lung fibroblasts, indicating that TBK1 may regulate the activation of NF-kB and the transcription of lfTSLP during hMPV infection. Overall, the TLR3 and RIG-I signaling pathways seem to converge on TBK1 to activate NF-κB-regulated expression of lfTSLP.

In summary, our study demonstrates that hMPV infection induces a robust expression of lfTSLP, but not sfTSLP, in human airway cells. The induction depends on NF-κB activation downstream both TLR3 and RIG-I and is facilitated by TBK1. This study reveals the molecular details of how hMPV elicits a pro-inflammatory TSLP response. However, since our data are derived from *in vitro* experiments, caution is needed in extending the interpretation of our results to a more complex physiological setting. How virus-mediated lfTSLP expression may influence host immune response and asthma pathogenesis *in vivo* remains elusive and needs further investigation. To better understand the potential causative relationship between respiratory virus infections and development of asthma, it will also be important to assess the expression of lfTSLP both upon virus-triggered infant bronchiolitis and in school-age children with or without asthma.

## Materials and Methods

### Virus propagation and titration

Sendai virus (Cantell strain) was purchased from Charles River Laboratories. Recombinant hMPV RecNL/1/00 (A1) was kindly provided by B. van den Hoogen (Erasmus MC, Rotterdam). LLC-MK2 monolayers were inoculated with virus at MOI 0.01 in OptiMEM (Thermo Fisher) containing 2% FBS, 20 µg/mL gentamicin and 0.68 mM glutamine. Virus was harvested after 7–9 days, purified on a 20% sucrose cushion and resuspended in OptiMEM (2% FBS). Purified virus was serially diluted (log10) on monolayers of LLC-MK2 cells in 96-well plates. Cells were washed after 4 days, stained with LIGHT DIAGNOSTICS™ hMPV direct fluorescence assay (Merck Millipore) and foci forming units were determined by manual counting.

### Cell culture and *in vitro* infection or interferon treatment

LLC-MK2 cells were cultivated in supplemented OptiMem (5% FBS, 0.68mM L-glutamine, 20 µg/mL gentamicin). A549 cells were cultivated in supplemented RPMI 1640 (10% FBS, 0.68 mM L-glutamine, 20 µg/mL gentamicin). BEAS-2B cells were cultivated in supplemented RPMI 1640 (10% heat-inactivated FBS, 0.68mM L-glutamine, 100U/ml penicillin, 100 µg/mL streptomycin). WI-38 fibroblasts were cultivated in supplemented DMEM (10% FBS, 100U/ml penicillin, 100 µg/mL streptomycin). TBK1 wild type and knockout immortalized murine embryonic fibroblasts (MEFs) were cultivated in supplemented DMEM (10% FBS, 0.68 mM L-glutamine, 100U/ml penicillin, 100 µg/mL streptomycin). Human monocyte-derived macrophages were derived from peripheral blood mononuclear cells (PBMCs) isolated from fresh buffy coats of healthy donors (blood bank of St. Olavs Hospital, Trondheim) using gradient centrifugation with Lymphoprep™ (Axis-Shield). PBMCs were seeded in RPMI 1640 medium (supplemented with 0.34 mM L-glutamine and 10 µg/mL gentamicin). After 2 h non-adherent cells were removed and adherent monocytes were cultivated in RPMI 1640 medium supplemented with 10% human serum (heat inactivated), 0.34 mM L-glutamine, 10 µg/mL gentamicin and 10 ng/mL M-CSF (Biolegend) for macrophage differentiation. Macrophages differentiated for 12–4 days were used in this study. All cells were incubated at 37 °C in 5% CO_2_ except MEF cells which were incubated in 8% CO_2_. Cells were inoculated with UV-inactivated or infectious hMPV in serum-reduced medium (2% FBS) at MOI 1, or SeV at hemagglutination units (HAU) 100, unless indicated otherwise. Recombinant IFNβ was obtained from Tebu-bio and used at the working concentration of 1000 U/mL. Recombinant IFNλ1 was obtained from PeproTech and used at the working concentration of 1 µg/mL.

### Quantitative real-time PCR

RNA was isolated with the RNeasy mini kit (Qiagen) following the manufacturer’s protocol. cDNA was synthesized from isolated RNA using the qScript kit (Quanta) following the manufacturer’s protocol. Quantitative real-time PCR (qRT-PCR) was performed using Perfecta SYBR Green reaction mix (Quanta) and a StepOnePlus instrument (Life Technologies) with the temperature profile 95 °C for 20 s, 40 cycles at 95 °C for 3 s and 60 °C for 30 s as previously described^47^. Fold change in gene expression was calculated using the ΔΔCt-method normalized against GAPDH. Primer sequences are listed in Supplementary Table 1. Prior to qRT-PCR analysis, GADPH was verified as a suitable reference gene under hMPV infection with regard to primer efficiency and stability as described by Livak *et al*.^48^.

### RNA interference

siRNAs were purchased from Qiagen (AllStars Negative Control), Ambion (RIG-I, TLR3, TRIF, RelA, IRF3, TRAF6 and TBK1) and Santa Cruz (MDA5, Lgp2, MAVS) respectively. siRNA duplexes were reverse transfected into cells using Lipofectamine RNAiMAX (Thermo Fisher Scientific) siRNA transfection reagent according to the manufacturer’s instructions. Transfected cells were allowed to grow for another 48 hours (medium was replaced after 24 hours) before hMPV infection or mock treatment.

### Western Blot

Following treatment cells were washed once in PBS and lysed in lysis buffer (50 mM Tris-HCl, 150 mM NaCl, 10% Glycerol, 0,5% Triton X-100 and 2 mM EDTA) containing phosphatase and protease inhibitors (100 mM Sodium Fluoride, 1 mM Sodium Orthovanadate, 40 mM β-Glycerophosphate, 10 µg/mL Leupeptin, 1 µM Pepstatin A and 1 mM Phenylmethylsulfonyl fluoride). Protein extracts were separated by NuPAGE® Bis-Tris gels (Thermo Fisher Scientific) and dry blotting was performed using iBlot® Gel Transfer stacks Nitrocellulose Mini kit and iBlot® machine (Invitrogen). Primary human antibody (mouse) for phospho-NF-κB p65 (sc-136548) was purchased from Santa Cruz. Primary human antibodies (rabbit) for NF-κB p65 (#3034), phospho-IRF3 (#4961) and IRF3 (#4962) were purchased from Cell Signaling Technology. Household β-actin antibody (mouse) (A1978) was purchased from SIGMA-ALDRICH and used as a loading control. Secondary antibodies (IRDye® 800CW Goat anti-Mouse, IRDye® 800CW Goat anti-Rabbit, IRDye® 680RD Goat anti-Mouse) were purchased from LI-COR Biosciences. LICOR Odyssey imager was used as the scanning system and relative intensity of protein bands was quantified with ImageJ.

### ELISA

Supernatants from WI-38 cell culture were collected and the concentration of TSLP was determined using a TSLP ELISA kit (BioLegend) following the manufacturer’s protocol.

### Immunofluorescence and confocal fluorescence microscopy

A549 or WI-38 cells were fixed in PBS containing 4% paraformaldehyde (PFA) for 10 min on ice followed by permeabilization with 0.1% saponin for 30 minutes and blocking with PBS containing 10% FBS and 2.5% BSA for 60 min at room temperature. Primary antibody against hMPV nucleoprotein (N protein) (MAB80138, Millipore) was incubated overnight at 4 °C, followed by secondary antibody GAM 647 (Molecular probes) for 30 min at room temperature. Nuclear staining was achieved with DAPI (Thermo Fisher) staining for 10 min.

### Statistics

Results are shown as mean ± SD from 3 technical replicates. A two-sided P-value < 0.05 as determined by Student t-test was considered significant. All data shown are representative for at least 3 independent experiments unless indicated otherwise.

### Data Availability

All data that support the findings of this study are available from the corresponding author upon reasonable request.

## Electronic supplementary material

Supplementary information
