# Supplementary material for: Characterization of signaling pathways regulating the expression of pro-inflammatory long form thymic stromal lymphopoietin upon human metapneumovirus infection
Source: Sci Rep. 2018 Jan 17;8:883. doi: 10.1038/s41598-018-19225-0 (PMC5772477; doi:10.1038/s41598-018-19225-0)
Supplement: Supplementary file 1 — Supplementary information [file 41598_2018_19225_MOESM1_ESM.doc]

**Characterization of signaling pathways regulating the expression of pro-inflammatory long form thymic stromal lymphopoietin upon human metapneumovirus infection**

Youxian Li^1^, Cecilie Lund^1^, Ida Nervik^1^, Simon Loevenich^1^, Henrik Døllner^1, 2^, Marit W. Anthonsen^1^, and Ingvild B. Johnsen^1,*^

^1^Department of Clinical and Molecular Medicine, Faculty of Medicine and Health Science, Norwegian University of Science and Technology, Trondheim, 7491, Norway

^2^Children’s Department, St. Olavs University Hospital, Trondheim, 7030, Norway

^*^[ingvild.johnsen@ntnu.no](mailto:ingvild.johnsen@ntnu.no)

**Supplementary Fig. 1 – hMPV replication in A549, BEAS-2B and WI-38 cells.** Cells were infected with hMPV at MOI 1 for indicated hours. Expression of hMPV-specific nucleoprotein RNA (as an indicator of viral RNA replication) was assessed by qRT-PCR. Data are representative for at least 2 independent experiments. Error bars represent SD of 3 technical replicates. **p < 0.01, ***p < 0.001 (Compared to 6h samples). N.D.: not detected.


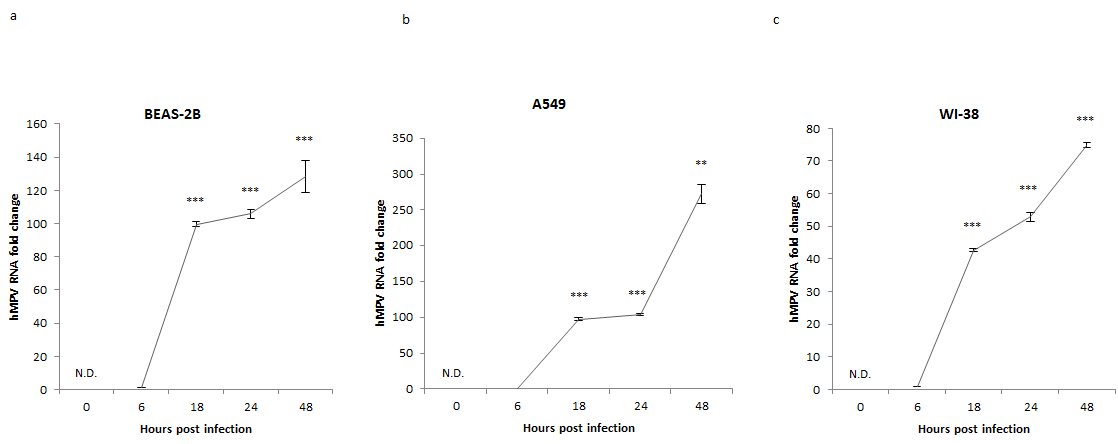


**Supplementary Fig. 2 – UV treated hMPV shows impaired replication and IFN-β induction**

WI-38 cells were infected with UV-inactivated hMPV or wild type hMPV for 18 hours. Expression of hMPV nucleoprotein RNA (a) or IFN-β mRNA (b) was assessed by qRT-PCR. Data are representative for at least 2 independent experiments. Error bars represent SD of 3 technical replicates. **p < 0.01, ***p < 0.001. NS: non-significant.


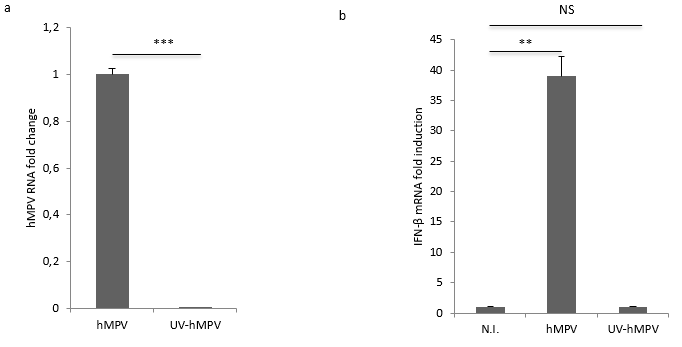


**Supplementary Fig. 3 – hMPV accumulates more rapidly in WI-38 cells**

Cells were infected with hMPV for 24 h. Prior to fixation the cells were incubated with DAPI (blue). Intracellular staining of hMPV N-protein (red) was performed prior to examination by confocal microscopy. Experiments were performed at least two times.


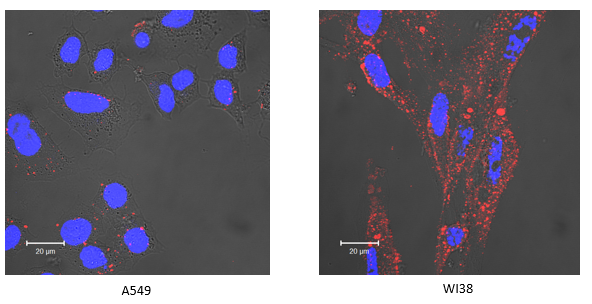


**Supplementary Fig. 4 – silencing efficiency of siRNAs**

Silencing efficiency of siRNAs was determined by qRT-PCR. Data are representative for at least 2 independent experiments. Error bars represent SD of 3 technical replicates. *p < 0.05, **p < 0.01, ***p < 0.001. (Compared to control siRNA transfection)


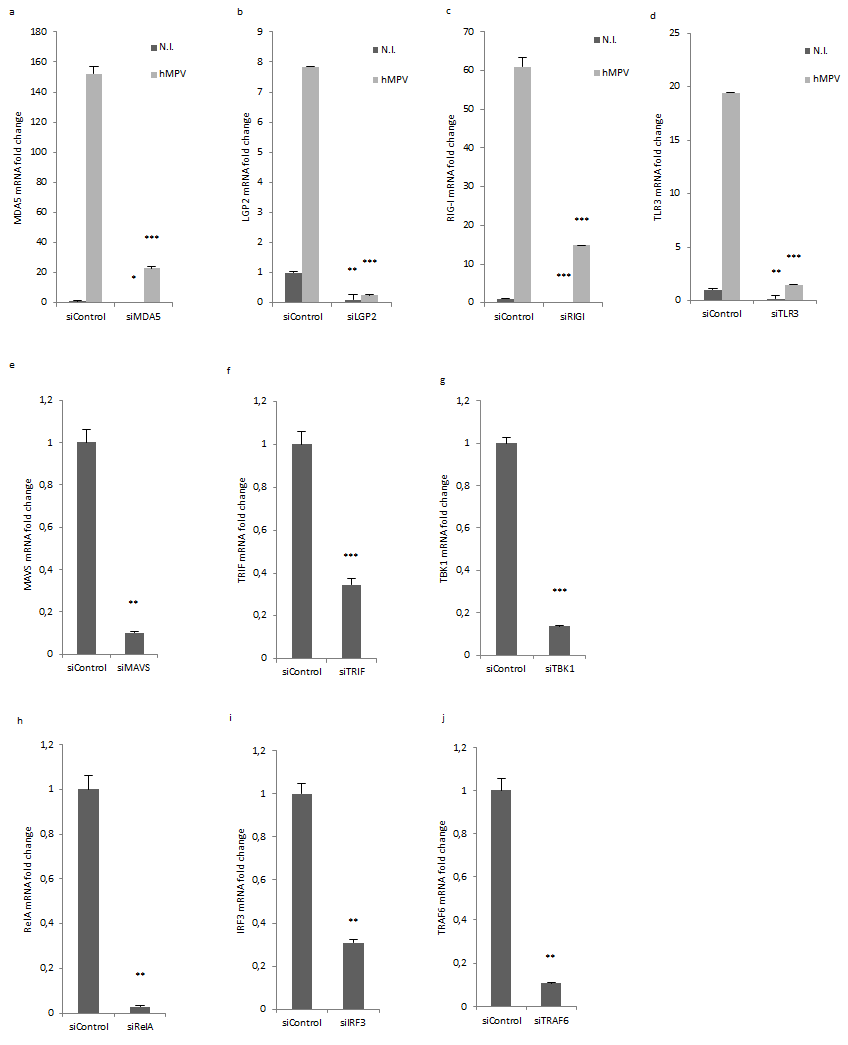


**Supplementary Fig. 5 – Type I or type III interferons do not induce lfTSLP expression.**

(a, b)WI-38 cells were treated with recombinant Interferon-β (rIFNβ) or infected with hMPV for 18 hours. lfTSLP (a) and OAS3 (b) mRNA expression was assessed by qRT-PCR. (c, d, e) WI-38 cells were treated with recombinant Interferon-λ1 (rIFNλ) or infected with hMPV for 18 hours. lfTSLP (c), OAS3 (d) and ISG54 (e) mRNA expression was assessed by qRT-PCR. (f, g) human monocyte-derived macrophages were treated with recombinant Interferon-λ1 (rIFNλ1) for 18 hours. OAS3 (f) and ISG54 (g) mRNA expression was assessed by qRT-PCR. Data are representative for at least 2 independent experiments. Error bars represent SD of 3 technical replicates. **p < 0.01, ***p < 0.001. (Compared to non-infected samples - N.I.)


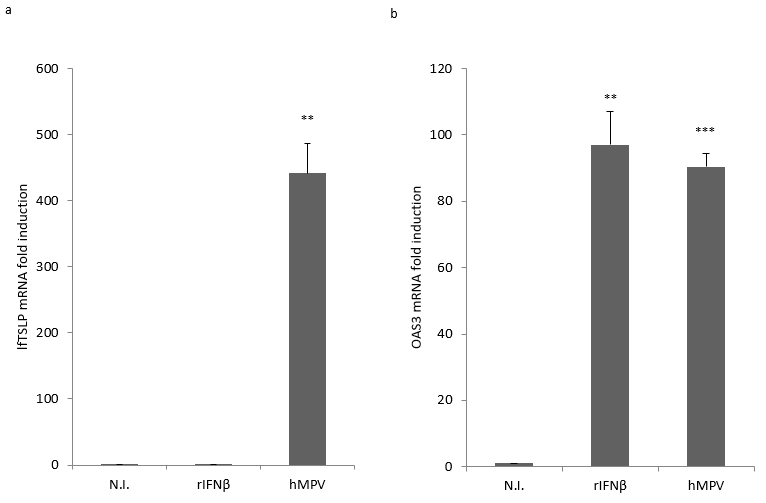


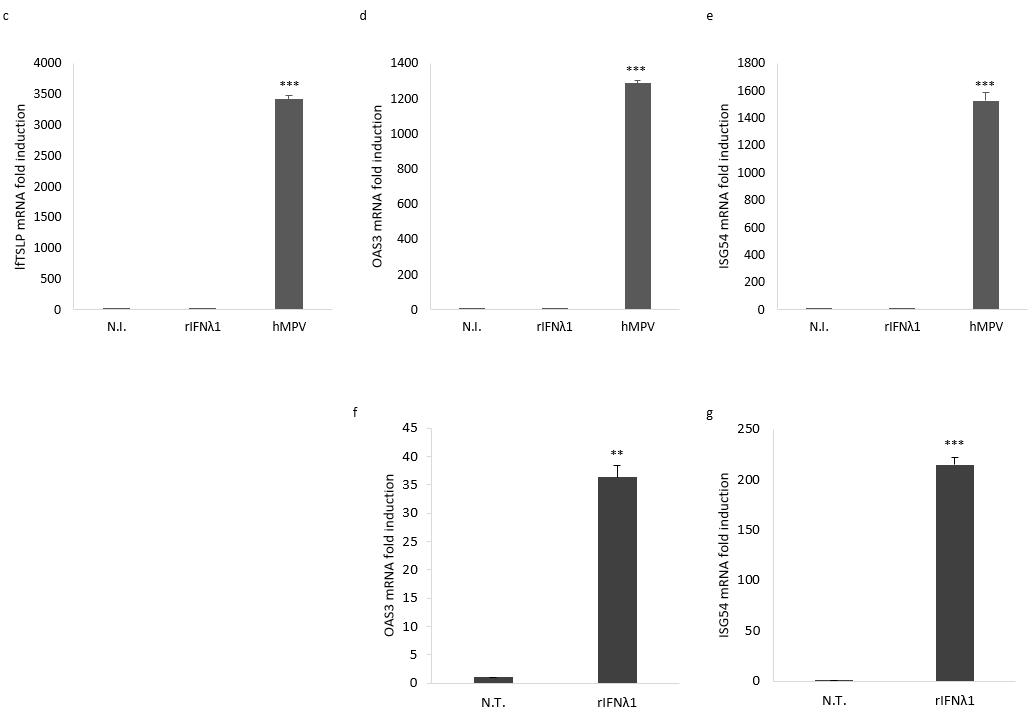


**Supplementary Table 1 – primers for qRT-PCR**

| Gene | Species | Forward | Reverse |
| --- | --- | --- | --- |
| lfTSLP | human | 5´-GGGCTGGTGTTAACTTACGACTTCA-3´ | 5´-ACTCGGTACTTTTGGTCCCACTCA-3´ |
| sfTSLP | human | 5´-CGTAAACTTTGCCGCCTATGA-3´ | 5´-TTCTTCATTGCCTGAGTAGCATTTAT-3´ |
| GAPDH | human | 5´-GAAGGTGAAGGTCGGAGTC-3´ | 5´-GAAGATGGTGATGGGATTTC-3´ |
| MDA5 | human | 5’-GGCACCATGGGAAGTGATT-3’ | 5’-ATTTGGTAAGGCCTGAGCTG-3’ |
| RIG-I | human | 5′-AGAGCACTTGTGGACGCTTT-3′ | 5′-TGCAATGTCAATGCCTTCAT-3′ |
| Lgp2 | human | 5´-ATGTGAACCCCAACTTCTCG-3´ | 5′-GACTTTGTTGATGACCACAGGA-3´ |
| TLR3 | human | 5´-AGAGTTGTCATCGAATCAAATTAAAG-3´ | 5´-AATCTTCCAATTGCGTGAAAA-3´ |
| MAVS | human | 5´-CCGTTTGCTGAAGACAAG-3´ | 5´-CTGGTAGCTCTGGTAGACAGAGGC-3´ |
| TRIF | human | 5´-AACAGAGCCAACACCTGGAC-3´ | 5´-GCTGAGTAGGCTGCGTTCA-3´ |
| RelA | human | 5´-TCATGAAGAAGAGTCCTTTCAGC-3´ | 5´-GGATGACGTAAAGGGATAGGG-3´ |
| IRF3 | human | 5´-AAGGAAGGAGGCGTGTTTG-3´ | 5´-TTCCTTCCGTGAAGGTAATCA-3´ |
| TRAF6 | human | 5´-TTTTGGTTGCCATGAAAAGA-3´ | 5´-CTCATGTGTGACTGGGTGTTC-3´ |
| TBK1 | human | 5´-TGTTTTGCGAGATGTGGTG-3´ | 5´-CTTCCCCTATAACACGCATGA-3´ |
| TSLP | mouse | 5´-AGGCTACCCTGAAACTGAG-3´ | 5´-GGAGATTGCATGAAGGAATACC-3´ |
| GAPDH | mouse | 5´-GGAAGGGCTCATGACCACA-3´ | 5´-CCGTTCAGCTCTGGGATGAC-3´ |
| IFN-β | human | 5´-GCCGCATTGACCATCTATGAGA-3´ | 5´-GAGATCTTCAGTTTCGGAGGTAAC-3´ |
| OAS-3 | human | 5´-TGCAGCGGCAGCTTAAGAGA-3´ | 5´-TGAGCATCCAGCACGTGGAA-3´ |
| ISG54 | human | 5´-TGGTGGCAGAAGAGGAAGAT-3´ | 5´-GTAGGCTGCTCTCCAAGGAA-3´ |
| hMPV | hMPV | 5´-CATATAAGCATGCTATATTAAAAGAGTCTC-3´ | 5´-CCTATTTCTGCAGCATATTTGTAATCAG-3´ |

Fig. 5a: Phospho-p65


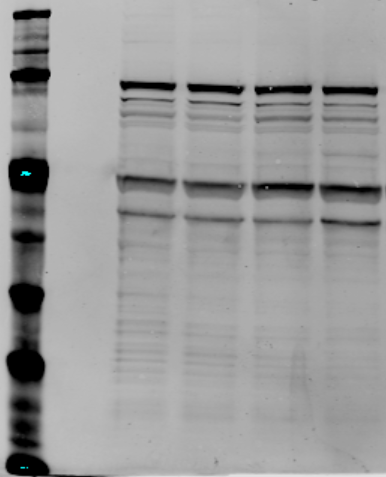


Phospho-p65

Fig. 5b: Phospho-IRF3 (same membrane as phospho-p65 above)


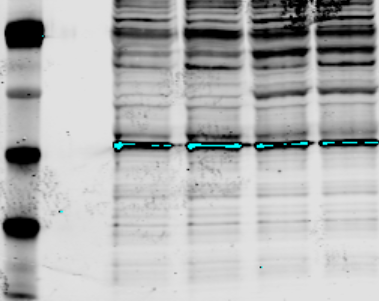


Phospho-IRF3

Fig. 5a,b: β-actin loading control (for phospho-p65 and phospho-IRF3)


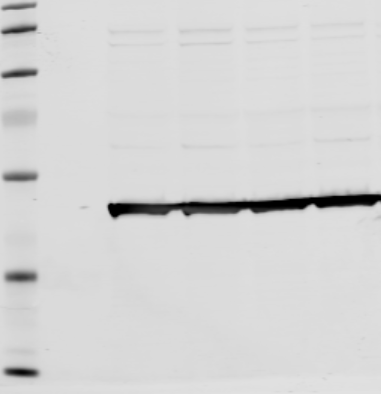


β-actin

Fig. 5a,b: p65 total and IRF3 total (same membrane)


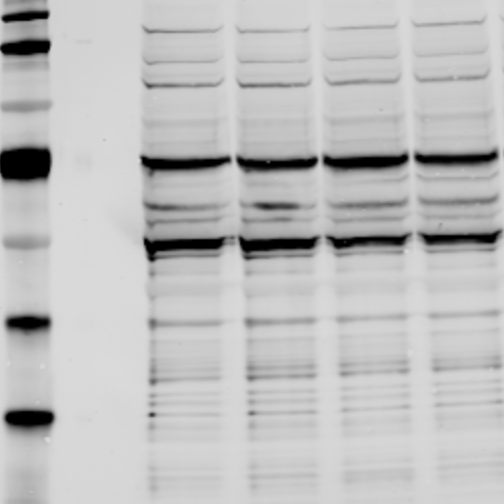


p65 total

IRF3 total

Fig. 5a,b: β-actin loading control (for p65 total and IRF3 total)


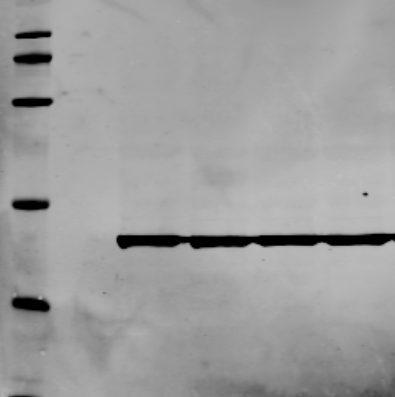


β-actin

Fig. 5g: phospho-p65


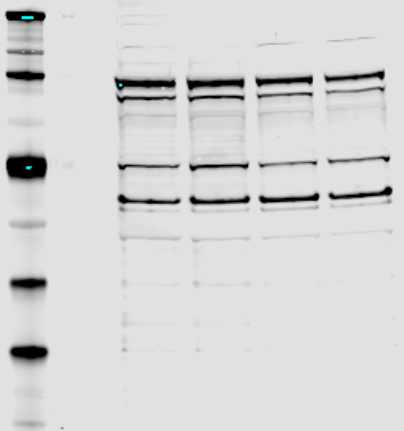


Phospho-p65

Fig. 5g: β-actin loading control (for phospho-p65)


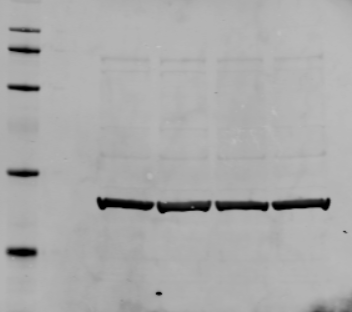


β-actin

Fig. 5g,h: p65 total and TBK1 (same membrane)


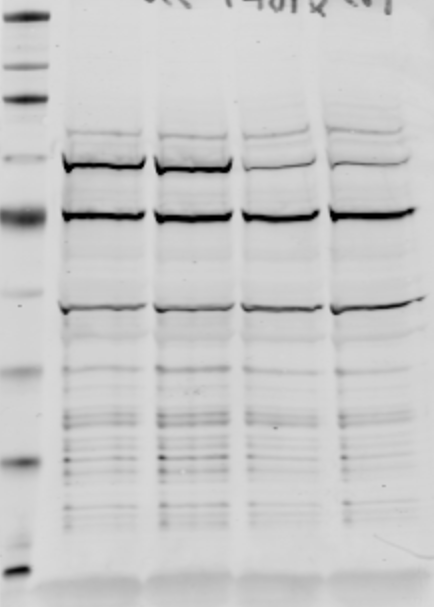


TBK1

p65 total

Fig. 5g,h: β-actin loading control (for p65 total and TBK1)


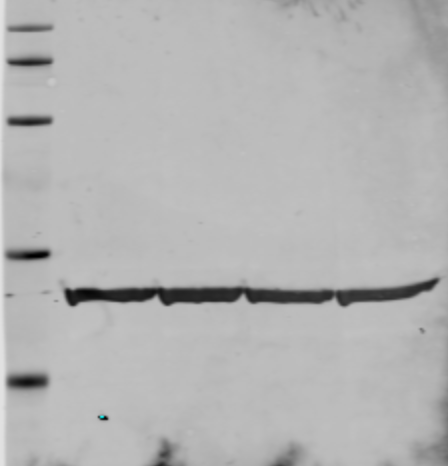


β-actin

Fig. 5h: phospho-IRF3


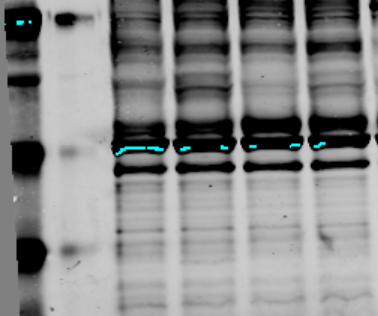


Phospho-IRF3

Fig. 5h: β-actin loading control (for phospho-IRF3)


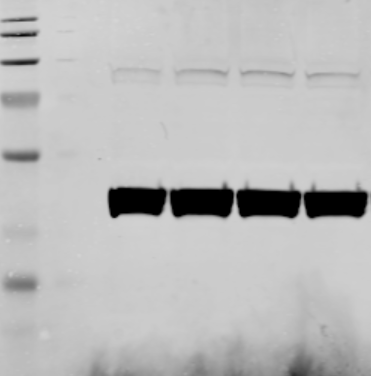


β-actin

Fig. 5h: IRF3 total


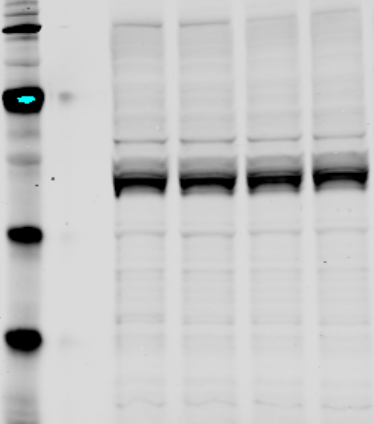


IRF3 total

Fig. 5h: β-actin loading control (for IRF3 total)


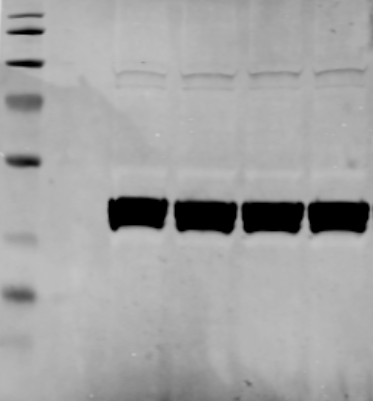


β-actin
